# A diverse suite of pharmaceuticals contaminates stream and riparian food webs

**DOI:** 10.1038/s41467-018-06822-w

**Published:** 2018-11-06

**Authors:** Erinn K. Richmond, Emma J. Rosi, David M. Walters, Jerker Fick, Stephen K. Hamilton, Tomas Brodin, Anna Sundelin, Michael R. Grace

**Affiliations:** 1 Water Studies Centre, School of Chemistry, Monash University, Clayton, 3800 Victoria Australia; 2 Cary Institute of Ecosystem Studies, Millbrook, NY 12545 USA; 3 U.S. Geological Survey, Fort Collins Science Center, Fort Collins, CO 80526 USA; 4 Department of Chemistry, Umeå University, Umeå, 90187 Sweden; 5 Kellogg Biological Station and Department of Integrative Biology, Michigan State University, Hickory Corners, MI 49060 USA; 6 Department of Ecology and Environmental Science, Umeå University, Umeå, 90187 Sweden; 7 Department of Wildlife Fish, and Environmental Studies, SLU, Umeå, 90187 Sweden; 8 Present Address: U.S. Geological Survey, Columbia Environmental Research Center, Columbia, MO 65201 USA

## Abstract

A multitude of biologically active pharmaceuticals contaminate surface waters globally, yet their presence in aquatic food webs remain largely unknown. Here, we show that over 60 pharmaceutical compounds can be detected in aquatic invertebrates and riparian spiders in six streams near Melbourne, Australia. Similar concentrations in aquatic invertebrate larvae and riparian predators suggest direct trophic transfer via emerging adult insects to riparian predators that consume them. As representative vertebrate predators feeding on aquatic invertebrates, platypus and brown trout could consume some drug classes such as antidepressants at as much as one-half of a recommended therapeutic dose for humans based on their estimated prey consumption rates, yet the consequences for fish and wildlife of this chronic exposure are unknown. Overall, this work highlights the potential exposure of aquatic and riparian biota to a diverse array of pharmaceuticals, resulting in exposures to some drugs that are comparable to human dosages.

## Abstract (web summary)

Pharmaceuticals are widespread contaminants in surface waters. Here, Richmond and colleagues show that dozens of pharmaceuticals accumulate in  food chains of streams, including in predators in adjacent terrestrial ecosystems.

## Introduction

Our exponentially increasing consumption of products containing a diversity of synthetic organic chemicals is accompanied by a concomitant increase in the diversity of chemical contaminants found in the environment, with uncertain ramifications for ecological and human health^1–3^. Pharmaceuticals and personal care products, which include a diverse array of organic chemicals widely used for public health, veterinary, and cosmetic and sanitary applications, are particularly likely to reach surface waters through wastewater inputs^4–7^. We have known for almost two decades that pharmaceuticals are widespread in surface waters^5^, yet the biological activity, exposure, and ecological effects of these contaminants in aquatic ecosystems remain poorly characterized^8–11^. Whereas some well-studied persistent organic contaminants, such as polychlorinated biphenyls (PCBs) and certain pesticides are known to accumulate in and may biomagnify through aquatic food webs^10,12^, we know very little about how individual pharmaceuticals accumulate in and affect organisms, or whether they may be biomagnified as they are transferred through food webs (but see ref. ^13^). Even less understood are the potential ecological effects of chronic exposures of biota to complex and variable mixtures of pharmaceuticals, yet this is the inevitable situation in surface waters influenced by wastewater inputs^14^.

In freshwaters, many species of aquatic insects have life cycles in which aquatic larvae emerge as terrestrial adults that fly above and away from the stream, serving as an important food resource for riparian predators, such as spiders, birds and bats^15^. This valuable ecological subsidy for riparian predators can, however, facilitate the movement of aquatic contaminants to terrestrial food webs. Walters et al.^16^ reported that a diverse assemblage of riparian insectivores, including spiders, accumulated PCBs originating in contaminated sediments via consumption of emerged adult insects. Biomagnification of organic contaminants such as PCBs through food webs can lead to toxic effects in animals higher up the food web, including top predators such as piscivorous birds and fishes, and, potentially, people who eat fish. Analogous, comprehensive studies of the fate of pharmaceuticals and their accumulation in food webs are lacking.

This study examines the fate of pharmaceutical contaminants within the aquatic biota as well as the inferred transfer of contaminants from emerging adult insects to riparian predators, thus employing a meta-community approach that is uncommon in the field of ecotoxicology^17^. Recent laboratory and field studies demonstrate that a diversity of pharmaceuticals can be found in aquatic animals and riparian consumers in water bodies receiving wastewater effluents^18^, but each of these studies focused on a limited number of species and chemicals^19–21^. Here, we present an assessment of 98 pharmaceuticals in aquatic invertebrates and riparian spiders collected from streams in Melbourne, Australia. We used these measurements to estimate the consumption of pharmaceuticals by two representative predators that feed almost exclusively on aquatic invertebrates—platypus (*Ornithorhynchus anatinus)*^22,23^ and brown trout (*Salmo trutta*)^22,24^. Our key findings are that a diverse suite of pharmaceuticals accumulates in aquatic invertebrates, and the similar concentrations and diversity of pharmaceuticals in riparian spiders demonstrate that pharmaceuticals in aquatic insect larvae are transferred to riparian predators via consumption of emerged adult insects. Moreover, our preliminary estimates suggest that platypus and brown trout, representatives of animals at the top of stream food webs, could in principle be exposed to certain drugs in their diets at levels comparable (up to 50%) to prescribed human doses.

## Results and Discussion

### Pharmaceuticals in aquatic food webs

**Fig. 1:**
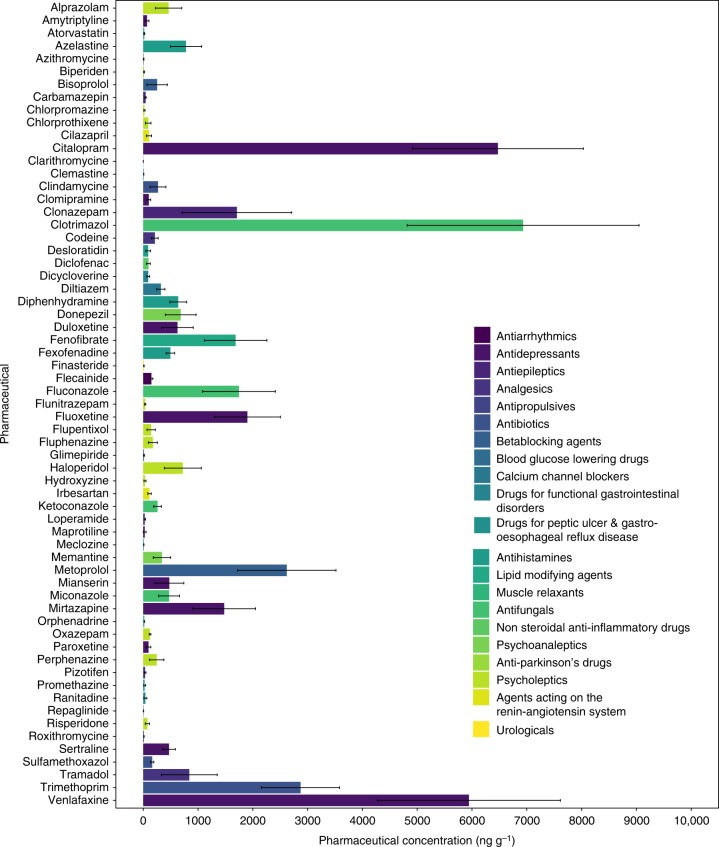
Pharmaceutical concentrations in caddisfly larvae. Mean pharmaceutical concentrations (ng g^−1^ dry weight ±1 SE) in caddisfly larvae (Hydropsychidae) (*n* = 6) at wastewater-influenced Brushy Creek. Each bar represents the mean concentration of a pharmaceutical compound in the six individuals collected over two sampling dates. Colours represent therapeutic drug classes

**Fig. 2:**
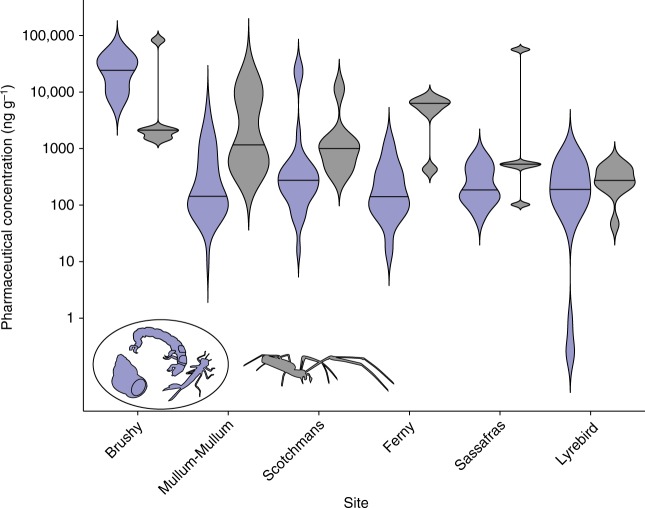
Pharmaceutical concentrations in benthic aquatic invertebrates and riparian spiders. Total pharmaceutical concentration (ng g^−1^ dry weight) in aquatic invertebrates (purple) and riparian web-building spiders (dark grey) for each study site, arranged in decreasing wastewater influence (indicated by δ^15^N in biofilms; Table 1). Violin plots illustrate kernel probability density and horizontal lines within each plot indicate median concentrations (see Methods for additional details on violin plots). The caddisfly image in this figure was adapted from Walters, D.M., M.A. Ford, and R.E. Zuellig. 2017. An open-source digital reference collection for aquatic macroinvertebrates of North America. Freshwater Science 36(4):693−697. DOI: 10.1086/694539. The spider image was adapted from a photo by Ryan R. Otter (Middle Tennessee State University)

We sampled over 190 aquatic insect larvae and other aquatic invertebrates from 18 taxonomic groups as well as riparian web-building spiders from in and around six streams in Melbourne, Australia. The streams span a gradient of wastewater inputs, including effluents from wastewater treatment facilities, leaking sewage infrastructure, and septic tanks, as indicated by enriched δ^15^N of stream biofilms^25^, which is characteristic of sewage nitrogen assimilation by stream primary producers^26^ (Table 1). We detected 69 pharmaceutical compounds from 23 therapeutic drug classes across all invertebrate taxa (Table 1), as exemplified by the filter-feeding caddisfly larvae of the Hydropsychidae family, which often dominate stream invertebrate communities (Fig. 1). Every invertebrate taxon tested had detectable concentrations, based on dry weight, of at least one pharmaceutical (i.e., >1 ng g^−1^) in its tissues, even though we included a reference site in Dandenong Ranges National Park (Lyrebird Creek) that we expected to be free of wastewater inputs. The highest pharmaceutical concentrations in aquatic invertebrates were found in or adjacent to a stream receiving effluent from a wastewater treatment facility (Brushy Creek) with tertiary treatment and disinfection. Concentrations of the sum of all pharmaceuticals in invertebrates from this site were typically 1–2 orders of magnitude higher on average than concentrations at other sites (Fig. 2, Table 1), except for Lyrebird Creek where concentrations were much lower. Across all sites, the top five most frequently detected pharmaceuticals in aquatic invertebrates were memantine, codeine, fluconazole, clotrimazol and mianserin (Supplementary Fig 1).

**Table 1:** Pharmaceutical concentrations detected in aquaitc invertebrates and spiders across six study sites

Site	δ^15^N biofilm (‰)	*n*	*P* (ng g^−1^)	SD	%RSD	Range (ng g^−1^)	*nP*	*nC*
Aquatic invertebrates								
Brushy	27.1 ± 3.3	28	27,205	16,994	62	4778–66,031	67	22
Mullum-Mullum	12.1 ± 2.48	44	566	983	174	10–5387	54	20
Scotchmans	10.6 ± 2.16	31	2548	7039	276	16–28,444	62	21
Ferny	10.2 ± 3.27	39	279	326	117	16–1265	55	21
Sassafras	9.2 ± 0.94	32	287	214	75	55–797	56	20
Lyrebird	7.7 ± 4.82	24	275	263	96	0.3–956	41	18
All sites		198	4506	11,545	256	0.3–66,037	69	23
**Riparian spiders**								
Brushy	—	6	15,347	32,993	215	1504–82,690	51	20
Mullum-Mullum	—	15	4786	5894	123	367–19,577	62	22
Scotchmans	—	8	2198	3776	172	320–11,490	41	16
Ferny	—	5	5084	2851	56	428–7747	56	19
Sassafras	—	5	11631	25,078	216	102–56,491	38	15
Lyrebird	—	10	313	157	50	46–564	31	16
All sites		49	5472	14,224	260	46–82,690	66	22

Average concentration of total pharmaceuticals (ng g^−1^ dry weight) and range of total pharmaceutical concentration detected in aquatic invertebrate and spider tissues across all sites. Elevated δ^15^N of the biofilms (*n* = 5) is an indicator of sewage contamination in streams

*P* average pharmaceutical concentration, *n* number of samples, *nP* number of pharmaceutical compounds, *nC* number of therapeutic classes, and *range* (ng g^−1^) minimum and maximum concentrations observed in biota

Emergence of adult insects from aquatic larvae and their consumption by predators provide a route for transfer of organic (e.g., PCBs and pharmaceuticals) and inorganic (e.g., heavy metals) contaminants from aquatic ecosystems to riparian and terrestrial food webs^27^. In this study, we detected 66 pharmaceutical compounds in riparian spiders that are known to be specialized consumers of emerged insects (Table 1, Fig. 2). At some sites, the concentrations of some pharmaceuticals in spiders were an order of magnitude higher than in aquatic invertebrates. For example, in spiders at Ferny Creek, concentrations of 19 pharmaceutical compounds were at least one order of magnitude higher than concentrations found in larval insects when excluding those with non-emergent life stages; these include anti-Parkinson’s drug memantine, analgesic tramadol, and the antihistamine diphenhydramine, among others from 12 therapeutic classes. This suggests that certain pharmaceuticals may biomagnify in food webs, but our data are inconclusive for two reasons. First, this pattern was not consistent across sites; concentrations were lower in spiders than larval insects at the most contaminated site (Brushy Creek). However, it is possible that insect emergence was limited at this site because it was the most impaired from an overall water quality perspective, and spiders there may have been more dependent on terrestrial food sources. Second, concentrations of highly bioaccumulative organic contaminants can be up to 3× higher in adult insects relative to their aquatic larvae because they lose considerable body mass during metamorphosis^28^. Thus the concentrations of pharmaceuticals in adult insects, which we did not measure, could be higher than the concentrations we measured in their larvae. Our data do indicate that pharmaceuticals in larval insect tissues were conserved through metamorphosis and that adult aquatic insects are a biological vector transporting pharmaceuticals to riparian predators, as has been shown for other contaminants^28^. Across all sites, the top five most frequently detected pharmaceuticals in riparian spiders were tramadol, codeine, fluconazole, metoprolol and clomipramine (Supplementary Fig 1). Our findings complement other research demonstrating that non-aquatic animals may be exposed to drugs originating from aquatic ecosystems^29,30^.

Aquatic invertebrates display a variety of different feeding modes, which might help to explain how each taxon differentially accumulated pharmaceuticals in their tissues. Filtering caddisflies (Hydropsychidae) that consume fine particulate material suspended in the water column contained the highest diversity (63 compounds) and concentrations of pharmaceuticals (Fig. 1) at the site downstream of a wastewater treatment facility. In contrast, at the same site, snails that consume algae growing on rocks had lower concentrations of pharmaceuticals; although, the diversity of compounds was similar (Supplementary Table 4). The differences we observed in accumulation of pharmaceuticals among invertebrates with different feeding modes, i.e. grazers feeding on algae, or invertebrates specialised to feed on particulate organic material, may provide an indication of how these contaminants are transferred to organic matter that forms the base of stream food webs.

**Fig. 3:**
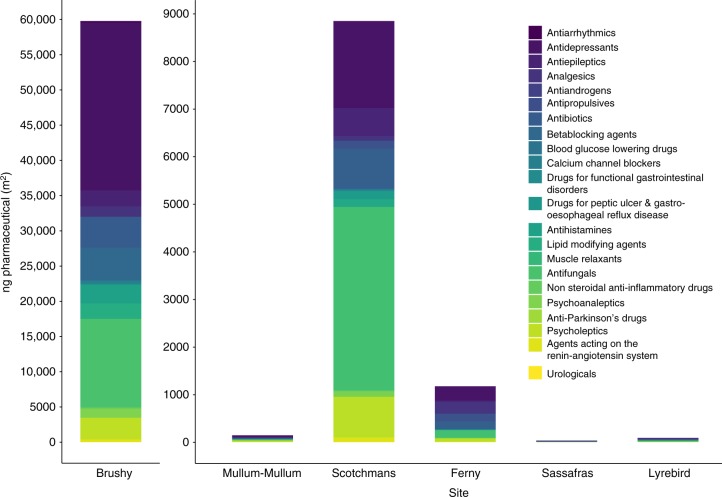
Proportions of pharmaceuticals in benthic aquatic invertebrates. Relative proportions of major classes of total pharmaceuticals (ng m^−2^ dry weight) in aquatic invertebrates at each site arranged by decreasing wastewater influence (indicated by δ^15^N in biofilms; Table 1). Each colour is representative of drug therapeutic class. Note data for wastewater-influenced Brushy Creek are on separate axis scale

We combined our data on invertebrate pharmaceutical concentrations with the biomass of invertebrates (including taxa with and without emergent adult stages) living in the stream (Fig. 3) to calculate the total pool of pharmaceuticals present in benthic stream organisms. The sum of pharmaceuticals in invertebrate tissues in the stream channel varied over 4 orders of magnitude among sites, with the highest amount at the stream receiving wastewater effluent (Brushy Creek: 60 µg m^−2^) and the lowest amount at forested and reference streams (Sassafras Creek: 32 ng m^−2^, Lyrebird Creek: 89 ng m^−2^). The relative abundances of different pharmaceutical classes also varied among sites; in wastewater-influenced Brushy Creek, antidepressants accounted for 40% by mass of the total pharmaceuticals in invertebrate biomass. Antidepressants were also high in invertebrate biomass at Mullum–Mullum Creek (25%, 37 ng antidepressants m^−2^) and at Ferny Creek (25%, 290 ng antidepressants m^−2^). In Scotchmans Creek, which has a high impervious surface cover of 42%^31^ typical of urbanized catchments, anti-fungal agents made up 43% (3900 ng anti-fungal agents m^−2^), while antidepressants contributed to 20% (1800 ng antidepressants m^−2^) of the total pharmaceutical pool of 8800 ng m^−2^ in invertebrate biomass.

### Associated risks for predators

In light of this evidence that a diverse suite of pharmaceuticals accumulates in the tissues of aquatic invertebrates, it is imperative to understand the risks that chronic exposure to these contaminants pose to predators, such as fishes and other wildlife, that rely on these invertebrates as their primary food resource. Risk analyses for persistent synthetic organic contaminants such as PCBs and chlorinated hydrocarbon pesticides have been conducted over many decades, supporting the development of aquatic-life benchmarks for concentrations of those contaminants^32,33^. Analogous benchmarks for pharmaceuticals are lacking, yet in many cases these compounds are expected to have biological effects, even at low concentrations, because they are pharmacologically active in humans or livestock. Many pharmaceutical compounds do have a recommended human daily dose for therapeutic measures, and this provides an approximate indication of thresholds for safe exposure in the absence of better information.

**Fig. 4:**
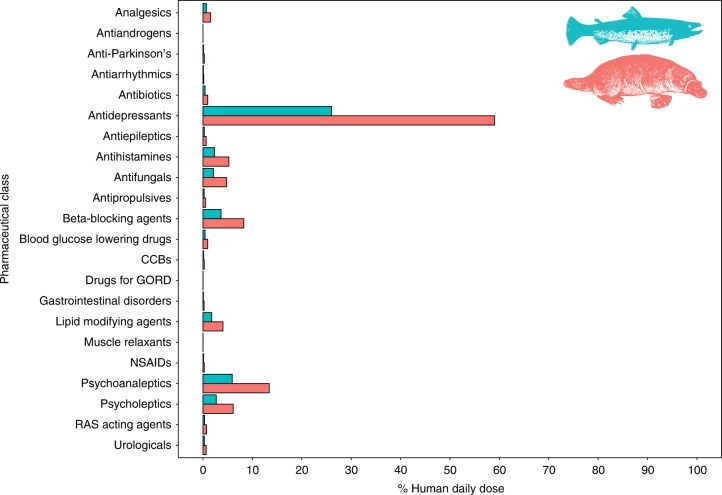
Estimated dietary intake of pharmaceuticals by two representative invertebrate predators compared to recommended human pharmaceutical doses. Dietary intake rates as a percentage of recommended human pharmaceutical daily doses by therapeutic class for platypus (pink) and brown trout (blue) in Brushy Creek. (CCBs calcium channel blockers, GORD gastroesophageal reflux disease, NSAID non-steroidal anti-inflammatory drugs, RAS renin angiotensin system). Calculations appear in Methods section (equations 2–5). The trout and platypus images in this figure were adapted from Harter, Jim. ‘Animals 1419 copyright-free illustrations of mammals, birds, fish, insects, etc. A pictorial archive from Nineteenth century sources’ Mineola, New York. Copyright Dover Publication Inc. (1979). All rights reserved

We used data from Brushy Creek, the site with wastewater inputs and the highest pharmaceutical concentrations in the invertebrates, to make comparisons between predator and human doses. We combined our data on the concentrations of pharmaceuticals per therapeutic class in aquatic invertebrates collected from Brushy Creek that corresponded to those found in benthic Surber samples, with modelled prey consumption rates to estimate pharmaceutical consumption by predators, then compared those rates to the average recommended daily doses per therapeutic class for humans^34^. As representative predators feeding mainly on aquatic invertebrates in Australian streams, we chose the native Australian platypus^22^ and non-native brown trout (Fig. 4), both of which occur in the study catchment^35–37^. We estimate, based on platypus energetics^38^, that a platypus consuming invertebrates from Brushy Creek would consume a total of 1154 µg kg^−1^ day^−1^ of pharmaceuticals spanning 67 compounds from 22 therapeutic drug classes. Platypus would thus consume about one-half of an average human daily dose of antidepressants by eating aquatic invertebrate prey from this stream (Fig. 4). Likewise, brown trout in Brushy Creek would consume a total of 509 µg kg^−1^ day^−1^ of pharmaceuticals, including 203 µg kg^−1^ day^−1^ of antidepressants, which equates to ~26% of a human daily dose (Fig. 4). Our estimates of prey consumption rates combined with contaminant concentrations in prey suggest that animals consuming aquatic invertebrates in streams with wastewater inputs would be exposed to a high diversity of biologically active compounds, and at doses for some drugs such as antidepressants that are of a similar magnitude as human therapeutic doses. The consequences for fish and wildlife of such chronic exposures to biologically active pharmaceuticals are unknown.

### Implications and future directions

We found complex mixtures of multiple and potentially interacting pharmaceuticals in stream invertebrate tissues, yet we screened our invertebrate tissue samples for only 98 compounds. This screening undoubtedly represents an underestimate of the diversity of compounds present in food webs because in the US market for example, there are over 1400 Food and Drug Administration (FDA) approved pharmaceuticals^39^; likewise, there are over 900 pharmaceuticals subsidized by the Australian Government’s Pharmaceutical Benefits Scheme^40^.

Although we do not yet understand the direct and indirect effects of these compounds, either singly or in complex mixtures, on fish and wildlife, a growing body of research demonstrates that many pharmaceuticals disrupt ecological interactions, functions and communities^11^. For example, amphetamines and antidepressants in stream water can disrupt the timing of emergence of aquatic insects^41,42^; psycholeptics such as Valium™ and the illicit drugs amphetamine and LSD can compromise the web-building ability of spiders^43^; and fish behaviour is altered when consuming prey contaminated with an antidepressant^44^. While pharmaceuticals have been detected in trace amounts (part per trillion concentrations) in surface waters around the world for over two decades, it was widely assumed that these concentrations pose little risk to the aquatic biota because environmental concentrations are usually well below lethal concentrations, and many of these compounds degrade rapidly in the environment, suggesting low risk of persistence and biomagnification potential (reviewed by^11^). In light of these assumptions, it was surprising that we detected such a diverse suite of pharmaceuticals in aquatic invertebrates and at such high concentrations (part per million concentrations of total pharmaceuticals in invertebrates at the most contaminated sites). Furthermore, the detection of similarly diverse pharmaceuticals at high concentrations in riparian spiders demonstrates that contamination is not limited to the stream channel; as adult insects emerge, they transfer pharmaceuticals into adjacent ecosystems. This movement may pose a risk to other terrestrial consumers of adult aquatic insects that rely heavily on aquatic insects as a food resource, such as frogs, bats and birds. By our estimates, predators that consume aquatic invertebrates in wastewater-influenced streams may be exposed to about one quarter and up to one-half of a human dose of some pharmaceuticals.

Human use of pharmaceuticals and personal care products is projected to increase with growing human populations^45^, and thus contamination of aquatic ecosystems and their food webs will continue to increase. More research is needed to understand how these complex mixtures of contaminants are affecting aquatic and adjacent riparian food webs.

## Methods

### Study sites and site characteristics

We selected 50-m reaches within six streams in the greater Eastern Melbourne region (Supplementary Table. 1), Victoria, Australia. Sites spanned a gradient of wastewater influence based on isotopic sewage signature, with multiple potential sources, including direct discharge from a wastewater treatment facility (WWTF), septic tank effluent, and leaky sewage infrastructure. Wastewater influence was estimated from biofilm δ^15^N, the stable isotope ratio of ^15^N:^14^N in organic matter coating rock surfaces on the stream bottom. Elevated δ^15^N is commonly used as an indicator of anthropogenic inputs to aquatic systems, particularly human derived waste^26^. Sites ranged in wastewater influence from a pristine site (Lyrebird Creek, Dandenong Ranges National Park) where no known sewage influence is present, to an urbanized stream (Brushy Creek) that receives treated sewage from a WWTF at a mean discharge rate of 15 ML d^−1^. Riparian habitats at the sampling reaches ranged from dense Eucalyptus forest (pristine site) to a mixture of mature trees, vegetation, and structures such as bridges (semi-urban sites), to revegetated patchy habitats with engineered channel walls (urban sites). We sampled each site twice over a 7-month period from June 2014 to January 2015.

### Assessment of stream physicochemical properties

At each site, physicochemical properties were characterised including flow and water chemistry (Supplementary Table 2). We collected unfiltered water samples in 50-mL polypropylene (PPE) bottles in triplicate for total nitrogen and total phosphorus analysis. We also collected triplicate water samples for ammonium (NH_4_^+^ + NH_3_), filterable reactive P (FRP), and NO_3_^−^ in 50-mL PPE bottles, after filtration through a 0.2 µm disposable sterile syringe membrane filter (Sartorius, Göttingen, Germany). Total organic carbon (TOC) samples (*n* = 3) were collected in pre-cleaned 50-mL amber glass jars. TOC samples were acidified with 2M HCl upon collection, before being refrigerated prior to analysis. Dissolved organic carbon (DOC) samples were also collected and acidified, after filtration through a membrane filter (pore size: 0.2 µm, diameter: 47 mm, PALL Corporation). We also collected dissolved inorganic carbon (DIC) samples. All water chemistry samples were analysed by the NATA-accredited Water Studies Centre Analytical Laboratory, Monash University, Melbourne (Australia). Other water quality variables were measured using a pre-calibrated Horiba U-10 Water Quality Checker. Variables measured in the field included pH, dissolved oxygen (DO) concentration, temperature, electrical conductivity (EC), and turbidity. Depending on the individual site we also measured flow either by the salt slug method^57^ or with a Pygmy Flow Meter (Hydrological Service Pty. Ltd., Australia).

### Algal stable isotope sample collection and analysis

The stable isotope ratio of biofilms (δ^15^N) was measured as an indicator of wastewater influence. At each site, 5 rocks, uniform in size, were randomly sampled and placed into zip-lock bags in the dark for transportation back to the laboratory. Rocks were then scrubbed to create a biofilm slurry from which subsamples were taken and dried at 60 °C. Biofilm samples were then ground to a fine powder and each sample was weighed and packed into tin capsules for analysis. Samples were analysed for nitrogen stable isotope ratios at the Water Studies Centre, Monash University on a calibrated elemental analyser (ANCA GSL2) interfaced to a continuous-flow isotope-ratio mass spectrometer (Hydra 20-22, Sercon Ltd., Cheshire, UK). Four internal standards (ammonium sulfate, sucrose, gelatine and bream) were used to correct for any variations in peak size linearity and instrumental drift. Typical precision was ±0.2 per mil for δ^15^N. Based on these internal standards, the accuracy of the data is calculated to fall within ±0.3 per mil for δ^15^N. All internal standards were calibrated against internationally recognised reference materials that included USGS 40, USGS 41, IAEA N1, USGS 25, USGS 26 and IAEA C-6 (the certified values can be obtained from https://nucleus.iaea.org). The laboratory checks the isotope values of internal standards every 6 months to ensure that there is no fractionation associated with the shelf life of the chemicals.

### Aquatic invertebrate and spider sample collection

To estimate benthic invertebrate population densities at each site, we collected 5 replicate quantitative benthic invertebrate samples using a Surber sampler (area 1200 cm^2^, mesh size 250 µm, Supplementary Table 5). Benthic samples were preserved on site with 70% ethanol for later identification in the laboratory. To estimate pharmaceutical concentrations in the total stream invertebrate community, we counted all individuals in Surber samples, estimated the biomass of individuals in those samples using the individual weights of each taxon measured in the isotope samples, and multiplied the biomass by the pharmaceutical concentrations in each taxon. We collected individual specimens from kick net samples (mesh size 500 µm, Wildco). From each study reach, we also collected web-building spiders (mostly from the family *Tetragnathidae*) before 07:00 h on the day of sampling by visually searching for webs on the banks within 1 m of the stream. Aquatic invertebrates were identified to genus or species where appropriate with reference to^46,47^. Spiders were identified to family level^48^. Identified invertebrates and spiders were rinsed with distilled water, any foreign material was removed, and specimens were frozen until pharmaceutical analysis (see below). The invertebrates analysed for pharmaceuticals accounted for over 90% of aquatic invertebrate biomass at all sites, with the exception of Sassafras and Lyrebird Creek, where due to higher community diversity but lower abundance of individual taxa, our samples were only able to account for 21 and 36% of the total biomass, respectively.

### Pharmaceutical analysis of invertebrate and spider tissue

We prepared frozen invertebrate specimens for pharmaceutical extraction by carefully placing single invertebrate and spider individuals in sterile microcentrifuge tubes, drying them for 24 h at 60 °C, and then weighing the dried samples on a microbalance. When invertebrates had low individual body mass (e.g., chironomids) we added additional individuals to the sample to create a composite to meet the minimum mass required for extraction (2 mg). Samples were placed in new sterile microcentrifuge tubes and were extracted after addition of 50 ng of a mixed internal pharmaceutical standard, and three extractions were performed: 1.5 mL methanol and water (7:3) with 0.1% formic acid, 1.5 ml acetonitrile, and 1.5 ml acetonitrile. Details of the extraction, pre-concentration through solid-phase extraction, and subsequent chemical analysis are given in ref. ^49^. Samples were analysed using a triple-stage quadrupole mass spectrometer (Quantum Ultra EMR (Thermo Fisher Scientific, San Jose, CA)) coupled with a liquid chromatographic pump (Accela, Thermo Fisher Scientific) and an autosampler (PAL HTC, CTC Analytics AG, Zwingen, Switzerland). See Supplementary Table 3 for a list of target pharmaceuticals and LOQ (limit of quantification) values.

### Limits of quantification

A result lower than the limit of quantification (LOQ) does not mean that the target analyte was not present, since the LOQ is the lowest concentration at which the compound can be reliably measured^50^. In order to develop an appropriate average concentration that included low concentrations, we estimated the concentrations for samples below detection. Within a taxon, if an individual sample was below the detection limit but another individual from that taxon and site had a measurable concentration of that pharmaceutical, we assigned the one that was below detection with half of the LOQ value. This is a standard technique to estimate concentrations below the detection limit^51^.

### QA/QC information for pharmaceutical analysis

For quality assurance and quality control (QA/QC), two MS/MS transitions were used to ensure positive identification of analytes with the criterion that the ratio between the transitions was not allowed to deviate more than ±30% from the ratio in the corresponding calibration standard within the same concentration range. Retention times for all analytes had to be within ±2.5% of the retention time in the corresponding calibration standard. Limits of quantification (LOQ) were determined from standard curves based on repeated measurements of low-level spiked tissue samples, and the lowest point in the standard curve that had a signal/noise ratio of 10 was considered to be equal to the LOQ. A seven-point matrix-adjusted calibration curve over the range of 0.05–100 ng ml^−1^ was used for linearity evaluation and quantification. Carry-over effects were evaluated by injecting standards at 100 ng l^−1^ followed by two mobile phase blanks. Several instrumental and field blanks were included in each analytical run.

### Data analysis and calculations

We used violin kernel probability density plots to display invertebrate and spider tissue concentrations (Fig. 2). Violin plots are a combination of a box plot and a kernel density plot. Each plot adds distribution data to summary statistics found in box plots, allowing for visual data analysis and exploration^52^. Where direct comparisons were made between invertebrates and riparian spiders, we included only insects with flying adult life stages.

The calculations of pharmaceuticals in invertebrates and spiders are shown in the next two subsections:

### Invertebrate enumeration and biomass estimation

The number and mass of benthic invertebrates per m^2^ of stream bottom were determined by equations 1–3. We averaged measured densities from Surber samples (*n* = 5 per site) from both sampling trips to estimate aquatic invertebrate abundances. The mass per individual was estimated from the total weight and number of individuals that contributed to the pooled dried samples used for pharmaceutical analysis. Equation 1 calculates the average biomass per individual in each pharmaceutical invertebrate sample:

1 $$M = x/i,$$

where $M$= biomass per individual (mg), $x$= total biomass of that taxon in the pharmaceutical sample (mg), and $i$= number of individuals per pharmaceutical sample.

The average biomass per individual, AM, was then arithmetically averaged overall replicate pharmaceutical samples (*n)* for a particular taxon, according to Equation 2:

2 $${\rm AM} = \mathop {\sum }\nolimits_1^n M/n.$$

The average number of individuals of that species found in a Surber sample, $n^{{\rm Surber}}$, was determined by the arithmetic mean of the number of individuals found in each of the *n* Surber samples at that site. Equation 3 then estimated the average biomass of that species, AveMass, per m^2^ of benthic invertebrate biomass:

3 $${\rm AveMass}\,({\rm mg}/{\rm m}^2) = n^{{\rm Surber}} \times {\rm AM} \times 10,000\,{\rm cm}^2/1200\,{\rm cm}^2.$$

### Pharmaceutical concentration estimates

For each taxon at each site, the average individual pharmaceutical concentration, AvePharm, per sample was determined per g of dried biomass according to Equation 4:

4 $${\rm AvePharm}\,({\rm ng}/{\rm g}) = \mathop {\sum }\nolimits_1^n {\rm Pharm}\,({\rm ng}/{\rm g})/n.$$

On an areal basis, the amount of each pharmaceutical per species per m^2^ of benthic invertebrates, ArealPharm, was given by Equation 5:

5 $${\rm ArealPharm}\left( {\frac{{{\rm ng}}}{{{\rm m}^2}}} \right) = {\rm AvePharm}\left( {\frac{{{\rm ng}}}{{\rm g}}} \right){\mathrm{x}}\frac{{{\rm AveMass}\left( {\frac{{{\rm mg}}}{{{\rm m}^2}}} \right)}}{{1000\left( {\frac{{{\rm mg}}}{{\rm g}}} \right)}}.$$

The areal pharmaceutical estimates were summed across all invertebrate taxa to give the total concentration of that pharmaceutical in benthic invertebrates per m^2^. Finally, all of the individual total pharmaceutical concentrations were summed to give the grand total of all pharmaceuticals in the benthic invertebrates per m^2^.

### Estimating pharmaceutical dietary intake by predators

Our estimates of the dietary intake of pharmaceuticals by platypus are based on a study of the macroinvertebrate prey of the platypus in the Shoalhaven River, New South Wales, Australia^38^. Energetic requirements of platypus have been estimated at 684^53^ and 341 kJ kg day^−^^1^^22^. For the purpose of this study, we took the mean of those two estimates (512.5 kJ kg day^−1^) and converted kJ kg to g kg (1 g of dry mass = 21 kJ), obtaining a daily food demand (as dry weight of invertebrates) of 24.4 g day^−1^. Based on foraging behaviour, a previous study estimated that platypus have a digestion efficiency of 80%^38^, and therefore to meet their metabolic needs, a platypus would need to eat 30.5 g kg^−1^ day^−1^. Platypus body weight is based on the mean weight of male and female platypus sampled in the Shoalhaven River (1.18 kg)^54^.

Brown trout dietary intake estimates are based on a previous estimate that at 15 °C, a 100 g trout would consume 1.35 g day^-1^ dry weight of invertebrate food^55^. We used these estimates to calculate the dietary intake of pharmaceuticals for a 100 g trout feeding at 15 °C.

Based on these estimates of prey consumption and the pharmaceutical concentrations measured in invertebrate tissues in Brushy Creek, the stream receiving treated wastewater effluent, we estimated the maximum amount of pharmaceutical ingested by a consumer (µg g^−^^1^). We calculated the percentage of a human therapeutic dose of pharmaceuticals to which a platypus and a trout would be exposed through consumption of aquatic invertebrates in this stream. To estimate average human daily dose, we obtained the maximum daily dose (DD) for each pharmaceutical compound that we detected using data obtained from the World Health Organisation^34^, and then calculated the average daily dose for that pharmaceutical class.

Therapeutic classes were defined using the World Health Organization (WHO) Anatomical Therapeutic Chemical (ATC) codes^34^. Pharmaceuticals were grouped into classes that best defined their therapeutic outcome, using ATC classes from levels 2, 3, and 4. Levels represent the organ on which they are biologically active and the chemical, therapeutic and pharmacological characteristics of the compound. Levels 2, 3 and 4 represent the chemical/therapeutic/pharmacological subgroups^34^ and most accurately group each of the pharmaceuticals detected in this study without going into excessive detail. In order to make therapeutic classification clearer to the reader, we chose to use common class names rather than the ATC classification. For example, we classed all anti-bacterials (including quinolones, fluoroquinolones, macrolides and tetracyclines) as antibiotics. Similarly, we took this approach for antimycotics, including anti-infectives, labelling them as anti-fungal medication. Supplementary Table 3 provides a summary of ATC classification and human daily dose. We also acknowledge that some compounds have multiple therapeutic uses, and have made reference to these compounds within the summary table.

Different aquatic invertebrates had different pharmaceutical concentrations in their tissues so the distribution of invertebrate taxa within the total benthic invertebrate biomass was used to estimate the biomass-weighted mean pharmaceutical concentrations of the benthic community as a whole. To account for the relative biomass of each invertebrate group in the stream (Brushy Creek), we used the following equation:

6 $${\rm fAveMass}_i = \frac{{{\rm AveMass}_i\left( {\frac{{{\rm ng}}}{{{\rm m}^2}}} \right)}}{{\mathop {\sum }\nolimits_{i = 1}^{i = j} {\rm AveMass}_i\,\left( {\frac{{{\rm ng}}}{{{\rm m}^2}}} \right)}},$$

where $\mathop {\sum }\limits_{i = 1}^{i = j} {\rm {AveMass}}_i$ is the sum of each individual areal biomass for the *j* invertebrate group present. For example, larval caddisflies of the Hydropsychidae family accounted for 73% of total invertebrate biomass in Brushy Creek, and their concentration of fluoxetine (an antidepressant) was 1901ng/g. The biomass-weighted mean pharmaceutical concentrations for Brushy Creek invertebrates were used to estimate relative consumption by a predator at that site, effectively assuming that predators consume invertebrate taxa in proportion to their relative biomass in the stream.

To calculate the amount of pharmaceuticals per therapeutic class consumed by a trout or platypus we used bio-energetic estimates and body mass. For example, a platypus needs 30.5 g dry weight of invertebrate prey per kg per day, and average weight of platypus = 1.18 kg. The daily intake of pharmaceuticals by the platypus is given by Equation 7:

7 $${\rm {Intake}}\left( {{\rm {ng}}\,{\rm {day}}^{ - 1}} \right) = \mathop {\sum }\limits_{i = 1}^{i = j} \left\{ {{\rm {fAveMass}}_i\,{\times}\,{\rm {AvePharm}}\left( {\frac{{{\rm {ng}}}}{{\rm {g}}}} \right)} \right\}{\mathrm{x}}\,30.5\,x\,1.18,$$

which can also be expressed on a per kg platypus body weight basis:

8 $${\rm {Intake}}\,\left( {{\rm {ng}}\,{\rm {kg}}\,{\rm {day}}^{ - 1}} \right) = {\rm {Intake}}\,\left( {{\rm {ng}}\,{\rm {day}}^{ - 1}} \right)/1.18\,{\rm {kg}}.$$

Finally, to estimate what percentage of the average recommended human daily dose (aveDD) a predator is exposed to through dietary intake, we used equations 9 and 10, where an average adult human from Oceania weighs 74.1 kg^56^.

9 $${\rm {Human}}\,{\rm {pharmaceutical}}\,{\rm {consumption}}\,\left( {{\mathrm{\mu }}{\rm {g}}\,{\rm {kg}}\,{\rm {day}}^{ - 1}} \right) = {\rm {aveDD}}\,\left( {{\rm {mg}}} \right)/74.1\,{\rm {kg}} \times 1000.$$

10 $${\mathrm{\% }}\,{\rm {of}}\,{\rm {human}}\,{\rm {DD}} = \frac{{{\rm {Predator}}\,{\rm {consumption}}\,({\mathrm{\mu }}{\rm {g}}\,{\rm {kg}}\,{\rm {day}}^{ - 1})}}{{{\rm {Human}}\,{\rm {consumption}}\,({\mathrm{\mu }}{\rm {g}}\,{\rm {kg}}\,{\rm {day}}^{ - 1})}} \times 100.$$

## Electronic supplementary material

Supplementary Information

## Data Availability

The data supporting the plots within this paper and other findings of this study are available from the corresponding authors upon request.
