# Supplementary material for: A diverse suite of pharmaceuticals contaminates stream and riparian food webs
Source: Nat Commun. 2018 Nov 6;9:4491. doi: 10.1038/s41467-018-06822-w (PMC6219508; doi:10.1038/s41467-018-06822-w)
Supplement: Supplementary file 1 — Supplementary Information [file 41467_2018_6822_MOESM1_ESM.pdf]

Richmond et al.

A diverse suite of pharmaceuticals contaminates stream and riparian food webs

Supplementary information:

Authors:

Erinn K. Richmond[1*], Emma Rosi[2], David. M. Walters[3], Jerker Fick[4], Stephen K. Hamilton[2,5], Tomas Brodin[4], Anna Sundelin[4] and Michael R. Grace[1]

Affiliations:

[1]Water Studies Centre, School of Chemistry, Monash University, Clayton Victoria, Australia

[2] Cary Institute of Ecosystem Studies, Millbrook, New York 12545, USA

[3] U.S. Geological Survey, Fort Collins, Colorado 80526, USA

[4] Umeå University, Sweden

[5]Kellogg Biological Station and Dept. Integrative Biology, Michigan State University, Hickory Corners, Michigan 49060, USA

* Corresponding author: erinn.richmond@monash.edu

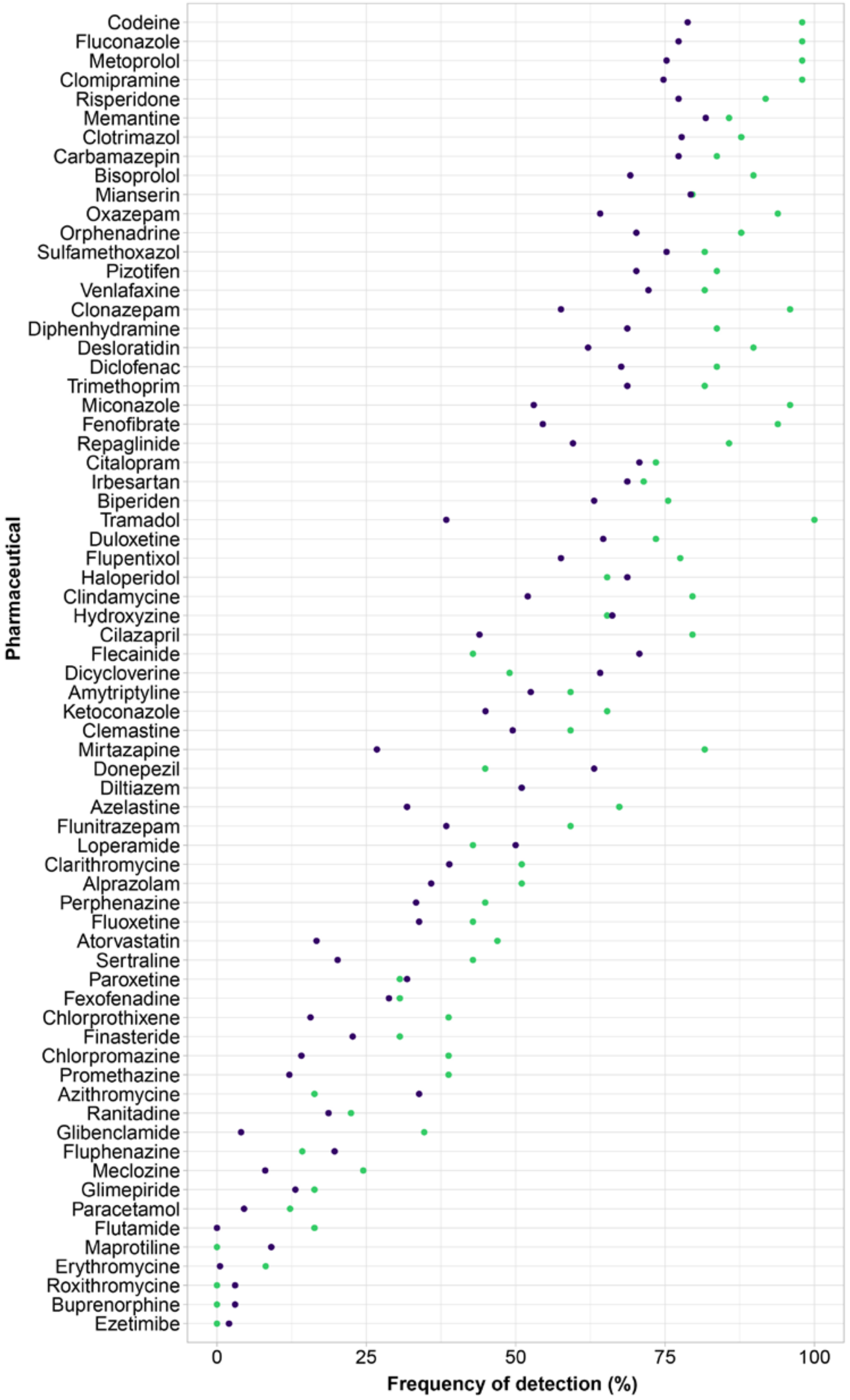

Supplementary Figure 1. Frequency of detections for larval insects with emergent adult stages (purple circles) and riparian spiders (green circles) across all six study sites arranged from most to least frequently detected.

Site	Suburb/ locality	Latitude/ Longitude coordinates
Brushy Creek	Churnside Park	-37.759363, 145.292702
Mullum-Mullum Creek	Donvale	-37.794465, 145.203102
Scotchmans Creek	Mount Waverley	-37.886777, 145.134560
Ferny Creek	Upwey	-37.915742, 145.325717
Sassafras Creek	Monbulk	-37.877138, 145.403029
Lyrebird Creek	Dandenong Ranges National Park	-37.850140, 145.384924

Supplementary Table 1. Location of study streams in eastern Melbourne, Victoria, Australia. Sites are ordered based on sewage influence (as indicated by biofilm $\delta^{15}N$) from highest to lowest

Supplementary Table 2. Physicochemical properties at all sites based on two sampling dates in 2014-2015. (EC = electrical conductivity mS/cm, Turb = turbidity in NTU (Nephelometric Turbidity Units), DO= dissolved oxygen, Temp = temperature, FRP = filterable reactive phosphorus, DOC = dissolved organic carbon, TOC = total organic carbon). SD = standard deviation. Water samples were collected and analysed in triplicate.

**2014**		**Flow (ML/d)**	**pH**	**EC (mS/cm)**	**Turb (NTU)**	**DO mg/L**	**Temp (^oC)**	**NH_3 mg/L**	**FRP mg/L**	**NO_2^- mg/L**	**NO_3^- mg/L**	**DOC mg/L**	**TOC mg/L**	**Total P mg/L**	**Total N mg/L**
Brushy	Mean	10.13	6.69	0.57	14.40	4.85	15.52	0.50	0.17	0.09	3.51	6.55	6.53	0.28	5.40
	SD.	-	0.01	0.00	0.55	0.05	0.04	0.01	0.00	0.00	0.10	0.03	0.04	0.01	0.36
Mullum-Mullum	Mean	1.50	7.23	0.31	19.20	10.12	10.10	0.03	0.03	0.01	0.88	4.12	4.22	0.08	1.40
	SD.	-	0.01	0.00	0.45	0.01	0.00	0.002	0.00	0.00	0.02	0.09	0.11	0.01	0.00
Scotchmans	Mean	22.91	6.59	0.24	22.00	8.27	12.60	0.03	0.02	0.01	0.61	5.51	6.38	0.08	1.53
	SD.	-	0.01	0.00	0.00	0.12	0.00	0.01	0.00	0.00	0.18	0.04	0.18	0.02	0.15
Ferny	Mean	1.94	5.77	0.13	13.40	10.51	9.81	0.02	0.02	0.01	1.09	8.69	11.21	0.07	1.67
	SD.	-	0.02	0.00	0.55	0.02	0.01	0.00	0.00	0.00	0.10	0.33	0.06	0.01	0.49
Sassafrass	Mean	6.19	5.77	0.13	13.40	10.51	9.81	0.01	0.05	0.00	1.81	1.63	1.92	0.07	2.30
	SD.	-	0.02	0.00	0.55	0.02	0.01	0.002	0.004	0.00	0.34	0.06	0.23	0.01	0.26
Lyrebird	Mean	2.82	7.02	0.88	39.60	9.29	11.60	0.03	0.003	0.01	0.40	4.64	6.12	0.04	0.80
	SD.	-	0.01	0.00	1.14	0.09	0.00	0.003	0	0.00	0.02	0.07	0.07	0.01	0.27

2015		Flow (ML/d)	pH	EC (mS/cm)	Turb (NTU)	DO mg/L	Temp (^oC)	NH_3 mg/L	FRP mg/L	NO_2- mg/L	NO_3- mg/L	DOC mg/L	TOC mg/L	Total P mg/L	Total N mg/L
Brushy	Mean	5.24	6.17	0.63	2.82	4.92	21.14	1.87	0.01	0.15	5.62	282	378.00	0.05	7.70
	SD.	-	0.00	0.00	1.58	0.06	0.00	0.06	0.00	0.01	0.32	286.4	101.39	0.00	1.48
Mullum-Mullum	Mean	0.87	8.37	0.30	6.20	10.72	22.61	0.05	0.03	0.01	0.26	42.3	44.67	0.07	0.67
	SD.	-	0.00	0.00	0.20	0.02	0.00	0.00	0.00	0.00	0.01	10.6	5.77	0.00	0.01
Scotchmans	Mean	0.43	7.06	0.45	0.00	3.99	17.68	0.10	0.02	0.01	0.10	24.33	43.33	0.09	0.50
	SD	-	0.00	0.00	0.00	0.01	0.00	0.00	0.00	0.00	0.00	6.35	22.37	0.00	0.05
Ferny	Mean	1.40	7.24	0.45	0.00	4.27	18.95	0.05	0.03	0.01	0.56	119.8	52.33	0.08	1.10
	SD.	-	0.00	0.00	0.00	0.01	0.00	0.00	0.00	0.00	0.06	33.26	40.53	0.00	0.00
Sassafras	Mean	3.62	6.69	0.13	4.91	5.57	16.80	0.09	0.37	0.01	0.52	20.5	16.67	0.26	2.43
	SD.	-	0.00	0.00	3.16	1.84	0.00	0.02	0.07	0.00	0.02	14.5	7.09	0.02	0.06
Lyrebird	Mean	0 .28	7.04	0.09	31.96	7.55	14.09	0.02	0.00	0.00	0.21	7.07	11.83	0.03	0.51
	SD.	-	0.01	0.00	0.23	0.02	0.00	0.00	0.00	0.00	0.00	0.46	3.88	0.00	0.03

Supplementary Table 3. *List of target pharmaceuticals tested, including ATC (anatomical therapeutic chemical) code, pharmaceutical name, therapeutic class, ATC level, maximum recommended defined daily dose for humans (Max DDD), and limit of quantification (LOQ). Footnotes denote additional therapeutic uses. Note that Max DDD per class was averaged to estimate what percentage dose a platypus or brown trout may be exposed to in this study.*

ATC Code	Pharmaceutical	Class	ATC Level	Max. DDD (mg/day)	LOQ (ng g^{1})
G04CA01	Alfuzosin	Urologicals	2	7.5	0.1
N05BA12	Alprazolam	Psycholeptics	2	1	10.0
C01BD01	Amiodarone	Antiarrhythmics	3	200	50.0
N06AA09	Amitriptyline	Antidepressants	2	75	5.0
C07AB03	Atenolol	Beta blocking agents	3	75	5.0
C10AA05	Atorvastatin	Lipid modifying agents	3	20	10.0
M03AC04	Atracurium	Muscle relaxants	3	ND	0.5
R01AC03	Azelastine	Antihistamines [1]	3	4	5.0
J01FA10	Azithromycin	Antibiotics [2]	4	500	5.0
R01AD01	Beclometasone	Corticosteroids [3]	4	1.5	10.0
N04AA02	Biperiden	Anti-Parkinson	2	10	0.1
C07AB07	Bisoprolol	Beta blocking agents	3	10	0.1
N04BC01	Bromocriptine	Anti-Parkinson [4]	2	40	5.0
A07EA06	Budesonide	Corticosteroids [3]	4	9	10.0
N07BC01	Buprenorphine	Opioids	3	8	10.0
N06AX12	Bupropion	Antidepressants	3	300	0.1
N03AF01	Carbamazepine	Antiepileptics	3	1000	1.0
N05AA01	Chlorpromazine	Psycholeptics	2	300	5.0
N05AF03	Chlorprothixene	Psycholeptics	2	300	10.0
C09AA08	Cilazapril	Agents acting on the renin-angiotensin system	2	2.5	1.0
J01MA02	Ciprofloxacin	Antibiotics [5]	4	1000	10.0
N06AB04	Citalopram	Antidepressants	3	20	5.0
J01FA09	Clarithromycin	Antibiotics	4	1000	1.0
R06AA04	Clemastine	Antihistamines	2	2	0.5
J01FF01	Clindamycin	Antibiotics [6]	4	180	1.0
N06AA04	Clomipramine	Antidepressants	3	100	0.5
N03AE01	Clonazepam	Antiepileptic	3	8	5.0
D01AC01	Clotrimazol	Antifungal	3	100	1.0
R05DA04	Codeine	Analgesics [7]	3	100	0.5
R06AX02	Cyproheptadine	Antihistamines	2	12	5.0
R06AX27	Desloratadine	Antihistamines	2	5	0.5
M01AB05	Diclofenac	NSAID	3	100	10.0

A03AA07	Dicycloverine	Drugs for functional gastrointestinal disorders	3	80	5.0
N02CA01	Dihydroergotamine	Analgesics	2	4	15.0
C08DB01	Diltiazem	Calcium channel blockers	2	240	0.5
R06AA02	Diphenhydramine	Antihistamines	2	300	0.1
B01AC07	Dipyridamole	Antithrombotic agent	3	400	1.0
N06DA02	Donepezil	Psychoanaleptics	2	7.5	0.5
N06AX21	Duloxetine	Antidepressants	3	60	1.0
C09CA02	Eprosartan	Agents acting on the renin-angiotensin system	2	600	5.0
J01FA01	Erythromycin	Antibiotics [2]	4	200	20.0
C10AX09	Ezetimibe	Lipid modifying agents	3	10	50.0
C10AB05	Fenofibrate	Lipid modifying agents	3	200	10.0
N01AH01	Fentanyl	Analgesics [8, 15]	2	1.2	0.5
R06AX26	Fexofenadine	Antihistamines	2	120	5.0
G04CB01	Finasteride	Urological [9]	2	5	10.0
C01BC04	Flecainide	Antiarrhythmics	3	200	0.1
J02AC01	Fluconazole	Antifungal	2	200	0.5
N05CD03	Flunitrazepam	Psycholeptics	2	1	10.0
N06AB03	Fluoxetine	Antidepressants	3	20	5.0
N05AF01	Flupentixol	Psycholeptics	3	6	5.0
N05AB02	Fluphenazine	Psycholeptics	3	10	10.0
L02BB01	Flutamide	Anti-Androgens	4	750	5.0
A10BB01	Glibenclamide	Blood glucose lowering drugs	3	10	10.0
A10BB12	Glimepiride	Blood glucose lowering drugs	3	2	10.0
N05AD01	Haloperidol	Psycholeptics	3	8	0.1
N05BB01	Hydroxyzine	Psycholeptics	3	75	0.5
C09CA04	Irbesartan	Agents acting on the renin-angiotensin system	2	150	0.5
J02AB02	Ketoconazole	Antifungal [10]	3	200	10.0
N05AA02	Levomepromazine	Psycholeptics	2	300	50.0
A07DA03	Loperamide	Anti propulsives	4	10	0.5
N06AA21	Maprotiline	Antidepressants	3	100	5.0
R06AE05	Meclizine	Antihistamines	3	50	5.0
N06DX01	Memantine	Psychoanaleptics	2	20	0.5
C07AB02	Metoprolol	Beta blocking agents	3	150	5.0
N06AX03	Mianserin	Antidepressants	3	60	1.0
D01AC02	Miconazole	Antifungal [11]	3	1000	5.0
N06AX11	Mirtazapine	Antidepressants	3	30	10.0
V03AB15	Naloxone	Antidotes	4	ND	1.0
N06AX06	Nefazodone	Antidepressants	3	400	0.5
J01MA06	Norfloxacin	Antibiotics [5]	4	800	10.0
J01MA01	Ofloxacin	Antibiotics [5]	4	400	10.0
N04AB02	Orphenadrine	Muscle relaxants	3	200	0.1
N05BA04	Oxazepam	Psycholeptics	2	50	5.0

J01AA06	Oxytetracycline	Antibiotics [13]	4	1000	10.0
N02BE01	Paracetamol	Analgesics	2	3000	10.0
N06AB05	Paroxetine	Antidepressants	3	20	10.0
N05AB03	Perphenazine	Psycholeptics	2	30	10.0
N02CX01	Pizotifen	Analgesics	2	1.5	0.5
R06AD02	Promethazine	Antihistamines	2	25	10.0
A02BA02	Ranitidine	Drugs for peptic ulcer and gastro-oesophageal reflux disease	3	300	5.0
A10BX02	Repaglinide	Blood glucose lowering drugs	3	4	0.5
N05AX08	Risperidone	Psycholeptics	2	5	0.1
C10AA07	Rosuvastatin	Lipid modifying agents	3	10	10.0
J01FA06	Roxithromycin	Antibiotics [2]	4	300	15.0
N06AB06	Sertraline	Antidepressants	3	50	10.0
C07AA07	Sotalol	Beta blocking agents	3	160	0.5
J01EC01	Sulfamethoxazole	Antibiotics [16]	4	2000	5.0
J01EE01	Tamoxifen	Endocrine therapy	2	20	5.0
L02BA01	Telmisartan	Agents acting on the renin-angiotensin system	2	40	1.0
C09CA07	Terbutaline	Adrenergics	3	20	0.5
R03CC03	Tetracycline	Antibiotics [13]	4	1000	50.0
J01AA07	Tramadol	Analgesics	2	300	5.0
N02AX02	Trihexyphenidyl	Anti-Parkinson	2	10	0.1
N04AA01	Trimethoprim	Antibiotics [14]	4	400	0.1
N06AX16	Venlafaxine	Antidepressants	3	100	0.5
C08DA01	Verapamil	Calcium channel blockers	2	240	10.0
N05CF02	Zolpidem	Psycholeptics	2	10	0.5

Table notes:

[1] Decongestants and anti-allergenic
[2] Macrolides
[3] Glucocorticoids
[4] Prolactin inhibitors
[5] Fluoroquinolones
[6] Lincosamide
[7] Cough suppressants
[8] Aesthetic
[9] Dermatological applications
[10] Gynaecological anti-infective
[11] Stomatological and topical
[12] Anti-Parkinson’s
[13] Tetracycline
[14] Trimethoprim derivatives
[15] Opioid
[16] Sulphonamides

Supplementary Table 4. Summary comparison of pharmaceutical concentrations (ng g^{-1}) in filter-feeding Hydropsychid caddisfly larvae and grazing snails (*Physa* sp.) at Brushy Creek. Total concentrations for the entire population and averages per individual are shown. *Note: Snail concentration is for tissue only; shells were removed.*

	Total pharm. Concentration (ng g^{-1})	Average pharm. Concentration (ng g^{-1})	± SE	Number of compounds
**Hydropsychid**	264,299	699	113	63
Physa	190,818	505	67	63

Supplementary Table 5. List of benthic invertebrate taxa at each site

Site	Order	Family
Brushy	Trichoptera	Hydropsychidae, *Cheumatopsyche*
	Trichoptera	Hydropsychidae, *Diplectrona*
	Trichoptera	Ecnomidae
	Trichoptera	Hydroptilidae
	Trichoptera	Hydrobiosidae
	Trichoptera	Conoesucidae
	Trichoptera	Leptoceridae
	Trichoptera	Helicophidae
	Trichoptera	Philorheithridae
	Trichoptera	Calocidae
	Diptera	Chironomidae, non-Tanypodinae
	Diptera	Chironomidae, *Tanypodinae*
	Diptera	Simuliidae
	Coleoptera	Psephenidae
	Physidae	Physidae
Mullum-Mullum	Trichoptera	Hydropsychidae, *Cheumatopsyche*
	Trichoptera	Ecnomidae
	Trichoptera	Hydrobiosidae
	Diptera	Chironomidae, non-Tanypodinae
	Diptera	Chironomidae, *Tanypodinae*
	Diptera	Simuliidae
	Diptera	Tipulidae
	Diptera	Psychodidae
	Ephemeroptera	Baetidae
	Coleoptera	Psephenidae
	Hemiptera	Corixidae
	Hemiptera	Velidae

	Physidae	Physidae
	Hygrophila	Planorbidae
	Pholadida	Sphaeriidae
	Oligochaeta	
Scotchmans	Trichoptera	Hydropsychidae, *Cheumatopsyche*
	Trichoptera	Ecnomidae
	Trichoptera	Hydroptilidae
	Diptera	Chironomidae, non-*Tanypodinae*
	Diptera	Chironomidae *Tanypodinae*
	Diptera	Simuliidae
	Odonata, Zygoptera	Megapodagrionidae
	Neotaenioglossa	Bithyniidae
	Physidae	Physidae
	Hygrophila	Planorbidae
	Hygrophila	Lymnaeidae
	Veneroida	Sphaeriidae
	Oligochaeta	
	Hirudinea	
Ferny	Trichoptera	Hydroptilidae
	Trichoptera	Hydrobiosidae
	Trichoptera	Leptoceridae
	Diptera	Chironomidae, non-*Tanypodinae*
	Diptera	Chironomidae, *Tanypodinae*
	Diptera	Simuliidae
	Diptera	Tipulidae
	Diptera	Ceratopogonidae
	Ephemeroptera	Baetidae
	Plecoptera	Gripopterygidae
	Coleoptera	Elmidae larva
	Coleoptera	Elmidae adult
	Hemiptera	Velidae
	Neotaenioglossa	Potamopyrgys
	Physidae	Physidae
	Hygrophila	Planorbidae
	Veneroida	Sphaeriidae
	Oligochaeta	
	Hirudinea	
	Nematoda	
Sassafras	Trichoptera	Hydropsychidae *Cheumatopsyche*
	Trichoptera	Hydrobiosidae
	Trichoptera	Conoesucidae
	Trichoptera	Leptoceridae
	Trichoptera	Helicophidae
	Trichoptera	Philorheithridae

	Diptera	Chironomidae, non-*Tanypodinae*
	Diptera	Chironomidae *Tanypodinae*
	Diptera	Simuliidae
	Diptera	Tipulidae
	Diptera	Tabanidae
	Diptera	Ceratopogonidae
	Diptera	Athericidae
	Ephemeroptera	Baetidae
	Ephemeroptera	Leptophlebiidae
	Plecoptera	Gripopterygidae
	Plecoptera	Austroperlidae
	Plecoptera	Notonemouridae
	Coleoptera	Psephenidae
	Coleoptera	Elmidae larva
	Coleoptera	Elmidae adult
	Coleoptera	Ptilodactylidae
	Coleoptera	Scirtidae
	Hemiptera	Velidae
	Mecoptera	Nannochoristidae
	Hygrophila	Planorbidae
	Hygrophila	Lymnaeidae
	Amphipoda	Eusiridae
	Oligochaeta	
	Isopoda	
Lyrebird	Trichoptera	Hydropsychidae *Cheumatopsyche*
	Trichoptera	Hydropsychidae *Diplectrona*
	Trichoptera	Hydrobiosidae
	Trichoptera	Philorheithridae
	Trichoptera	Calocidae
	Diptera	Chironomidae, non-*Tanypodinae*
	Diptera	Chironomidae *Tanypodinae*
	Diptera	Simuliidae
	Diptera	Tipulidae
	Diptera	Tabanidae
	Diptera	Ceratopogonidae
	Diptera	Dolichopodidae
	Ephemeroptera	Leptophlebiidae
	Ephemeroptera	Oniscigastridae
	Plecoptera	Gripopterygidae
	Plecoptera	Austroperlidae
	Plecoptera	Notonemouridae
	Coleoptera	Psephenidae
	Coleoptera	Elmidae larva
	Coleoptera	Elmidae adult

	Coleptera	Ptilodactylidae
	Odonata, Epiprocta	Corduliidae
	Odonata, Epiprocta	Aeshniidae
	Mecoptera	Nannochoristidae
	Megaloptera	Sialidae
	Veneroida	Sphaeriidae
	Amphipoda	Eusiridae
	Amphipoda	Talitridae
	Oligochaeta	
	Nematomorpha	Gordioidea
